# Smart Safety Helmets with Integrated Vision Systems for Industrial Infrastructure Inspection: A Comprehensive Review of VSLAM-Enabled Technologies

**DOI:** 10.3390/s25154834

**Published:** 2025-08-06

**Authors:** Emmanuel A. Merchán-Cruz, Samuel Moveh, Oleksandr Pasha, Reinis Tocelovskis, Alexander Grakovski, Alexander Krainyukov, Nikita Ostrovenecs, Ivans Gercevs, Vladimirs Petrovs

**Affiliations:** 1 Engineering Faculty, Transport and Telecommunication Institute, Lauvas Iela 2, LV-1019 Riga, Latvia; moveh.s@tsi.lv (S.M.); avg@tsi.lv (A.G.); krainukovs.a@tsi.lv (A.K.); ostrovenecs.n@tsi.lv (N.O.); gercevs.i@tsi.lv (I.G.); petrovs.v@tsi.lv (V.P.); 2 3D Engineering SIA, Mežu Iela 41-33, LV-3405 Liepaja, Latvia; oleksandr@3d-engineering.eu (O.P.); reinis@3d-engineering.eu (R.T.)

**Keywords:** VSLAM, smart, safety, helmet, vision, integrated

## Abstract

Smart safety helmets equipped with vision systems are emerging as powerful tools for industrial infrastructure inspection. This paper presents a comprehensive state-of-the-art review of such VSLAM-enabled (Visual Simultaneous Localization and Mapping) helmets. We surveyed the evolution from basic helmet cameras to intelligent, sensor-fused inspection platforms, highlighting how modern helmets leverage real-time visual SLAM algorithms to map environments and assist inspectors. A systematic literature search was conducted targeting high-impact journals, patents, and industry reports. We classify helmet-integrated camera systems into monocular, stereo, and omnidirectional types and compare their capabilities for infrastructure inspection. We examine core VSLAM algorithms (feature-based, direct, hybrid, and deep-learning-enhanced) and discuss their adaptation to wearable platforms. Multi-sensor fusion approaches integrating inertial, LiDAR, and GNSS data are reviewed, along with edge/cloud processing architectures enabling real-time performance. This paper compiles numerous industrial use cases, from bridges and tunnels to plants and power facilities, demonstrating significant improvements in inspection efficiency, data quality, and worker safety. Key challenges are analyzed, including technical hurdles (battery life, processing limits, and harsh environments), human factors (ergonomics, training, and cognitive load), and regulatory issues (safety certification and data privacy). We also identify emerging trends, such as semantic SLAM, AI-driven defect recognition, hardware miniaturization, and collaborative multi-helmet systems. This review finds that VSLAM-equipped smart helmets offer a transformative approach to infrastructure inspection, enabling real-time mapping, augmented awareness, and safer workflows. We conclude by highlighting current research gaps, notably in standardizing systems and integrating with asset management, and provide recommendations for industry adoption and future research directions.

## 1. Introduction

Aging infrastructure and complex industrial assets require regular inspections to ensure safety and reliability [1]. Traditional inspection methods are often labor-intensive, time-consuming, and potentially hazardous to personnel (e.g., climbing structures or entering confined spaces) [2,3]. There is a growing need for more efficient and safer inspection techniques. In recent years, wearable technology, particularly smart safety helmets with integrated sensors, has garnered attention as a means to augment inspectors’ capabilities and reduce risks [4,5,6]. These helmets serve as advanced personal protective equipment (PPE) that not only shield the worker but also embed cameras and other sensors to monitor and analyze the environment in real time. Industrial environments such as construction sites, oil and gas plants, and power generation facilities are high-risk settings where situational awareness is critical. Every year, thousands of workplace injuries and fatalities are recorded in such sectors, often during inspection and maintenance activities [7,8]. By providing hands-free visual monitoring and on-demand information, smart helmets aim to enhance safety and reduce human error [9].

Concurrently, infrastructure owners face increasing pressure to maintain aging bridges, tunnels, and factories with limited resources. Manual inspection techniques (visual checks, pen-and-paper note-taking, etc.) can be inefficient and may not detect subtle issues until they worsen [10]. Digital inspection technologies promise better coverage and data fidelity. Drones and robots have been used for remote inspection of hard-to-reach areas, but there remain scenarios where a human inspector’s presence and judgment are indispensable [11,12]; for example, examining complex machinery internals or performing detailed nondestructive tests onsite. In these cases, a smart helmet with an integrated vision system can empower the inspector by streaming a video to remote experts, overlaying augmented reality (AR) guidance, and simultaneously building a visual map of the inspected asset. Such capabilities improve inspection accuracy while minimizing exposure to hazards [13,14].

A particularly enabling technology for these advanced helmets is visual SLAM (Simultaneous Localization and Mapping). VSLAM algorithms allow a moving camera to map an environment and track its own position within that map [15,16,17]. Originally developed in robotics, VSLAM enables a helmet-mounted camera to function as the “eyes” of the inspector, continuously capturing the surroundings and creating a 3D model of structural elements (beams, pipes, walls, etc.) as the inspector moves [18,19]. This map can serve multiple purposes, guiding the inspector (by providing navigation cues in complex facilities or GPS-denied areas like mines), identifying structural defects via 3D point cloud analysis, and registering inspection data (photos, thermal images, measurements) to specific locations on the asset. The importance of VSLAM is underscored by its ability to work where GNSS fails in indoor or cluttered environments, offering a self-contained positioning method based on vision [20,21].

As cameras are lightweight, low-cost, and informative, they are increasingly favored over heavy, expensive LiDAR sensors for many applications [22]. Visual methods can thus dramatically augment infrastructure inspection capabilities, provided that robust algorithms and hardware are in place.

### 1.1. Research Objectives

This review aims to address the following research questions about VSLAM-enabled smart safety helmets for industrial infrastructure inspection.

Primary objective: to systematically analyze and evaluate smart safety helmets equipped with visual SLAM technologies, examining their technological capabilities, deployment effectiveness across industrial inspection scenarios, and implementation challenges to guide future research and industrial adoption.

Specific research questions:

RQ1 (Technology Characterization): how can current helmet-mounted vision and mapping technologies be examined and categorized, especially VSLAM algorithms and camera system configurations?

RQ2 (Performance Assessment): how well do these systems perform across different industrial inspection scenarios, and what quantifiable benefits have been demonstrated in real-world deployments?

RQ3 (Implementation Analysis): what are the key technical, human factor, and regulatory challenges that affect widespread adoption, along with identified best practices for successful deployment?

RQ4 (Future Directions): what are the current research gaps, emerging technological trends, and priority areas for future research development?

Review scope: This analysis focuses on peer-reviewed literature, patents, and industry reports spanning the last decade (2015–2025), particularly emphasizing the recent period when advances in wearable computing and visual SLAM have significantly accelerated. This review specifically targets helmet-integrated vision systems designed for industrial inspection applications, excluding purely consumer or recreational uses.

Target audience: this review is intended for researchers in computer vision and robotics, industrial engineers involved in infrastructure inspection, technology developers working on wearable systems, and decision makers evaluating smart helmet adoption for industrial applications.

The research questions are systematically addressed through the following paper structure: RQ1 (Technology Characterization) is examined in Section 3 (Camera System Typologies) and Section 4 (VSLAM Technologies and Implementations), where we analyze and categorize camera configurations, VSLAM algorithms, and sensor fusion approaches. RQ2 (Performance Assessment) is addressed in Section 5 (Industrial Applications and Use Cases), which evaluates system effectiveness across different inspection domains and presents quantified benefits from real-world deployments. RQ3 (Implementation Analysis) is covered in Section 6 (Challenges and Limitations), examining technical, human factor, and regulatory barriers, while Section 7 (Collaborative and Multi-agent Systems) explores advanced deployment approaches. Finally, RQ4 (Future Directions) is addressed in Section 8 (Conclusion and Recommendations), which synthesizes current research gaps and provides forward-looking guidance for technology development and industrial adoption.

### 1.2. Historical Evolution of Smart Helmet Technology

The concept of integrating cameras and sensors into helmets has evolved considerably over the past few decades. Early instances in the late 20th century were rudimentary, often driven by research in wearable computing and augmented reality (AR). In 1997, Steve Mann’s seminal work on wearable computing described a “wearable tetherless computer with video capability” as a first step toward personal imaging [23,24,25]. These initial prototypes were cumbersome, involving helmets or headgear connected to backpack PCs and basic cameras, yet they demonstrated the potential of hands-free recording and overlaying information onto a user’s view. Around the same time, the military and law enforcement experimented with helmet-mounted cameras for training and field documentation. However, these systems were largely for offline recording; the idea of real-time processing was limited by the computing hardware of the era.

The early 2000s saw significant developments. Notably, a 2004 U.S. patent by Hartwell and Brug described an “Integrated Smart Helmet” with built-in electronics for safety and convenience features [26]. This helmet included a GPS for location, sensors for environment interaction, a small display, and communications capability, essentially an early blueprint of a multi-sensor smart helmet [27]. Another landmark was the OmniVision helmet camera [28], which patented a completely integrated helmet camera unit with a single-chip CMOS sensor, wireless video transmission, and remote control features. By minimizing size and weight, this invention made it feasible to embed a camera directly in a helmet without encumbering the wearer, reflecting a shift toward truly wearable designs. Such innovations were primarily applied to sports (e.g., helmet cams for skydiving or motocross) and firefighting or policing (for incident recording). They laid the groundwork for treating the helmet as a sensor hub rather than just protection. In the late 2000s and early 2010s, parallel advances in computer vision and SLAM began to influence wearable systems.

On one hand, the computer vision community achieved real-time monocular SLAM on PCs [29]; for example, MonoSLAM was one of the first real-time camera SLAM systems, and PTAM (Parallel Tracking and Mapping) demonstrated robust AR tracking on a handheld device. On the other hand [30], wearable tech advanced with projects like Microsoft’s Wearable AR prototypes and early industrial smart glasses (e.g., Google Glass released in 2013, albeit more of a consumer device). The convergence was evident in research prototypes by 2011. The authors of [31] discussed the optimal placement of wearable vision sensors, including head-mounted stereo rigs. By 2012, a team led by Gutierrez-Gomez, Puig, and Guerrero [32] achieved full 3D visual odometry using an omnidirectional camera on a helmet. Their system recovered metric scale and produced 3D trajectories from a single wide-angle camera, showing that even without dedicated depth sensors, a helmet camera could perform SLAM in principle. Around the same time, the term “smart helmet” started appearing in the literature for systems that improve situational awareness. For instance, a 2013 study by [33] introduced a smart helmet for motorcyclists that could detect accidents and enforce helmet use (though not vision-based, it reflects growing interest in helmet-mounted intelligence).

The mid-2010s saw an inflection point with the emergence of robust VSLAM algorithms and the rise in industrial AR solutions. ORB-SLAM (2015) provided a versatile, high-accuracy monocular SLAM solution with loop closure [34], which was soon extended to stereo and RGB-D versions. Around this time, commercial enterprises brought smart helmets to the market: DAQRI introduced its smart helmet in 2014, integrating an Intel processor, multiple cameras (including a depth camera), and a transparent AR visor [35]. The device was specifically aimed at industry, enabling workers to see overlaid building plans and sensor data in real time, effectively allowing them to “see through” structures with thermal vision and X-ray-like visualization of hidden infrastructure. This marked a shift from using helmet cameras purely for recording or remote viewing to using them for real-time augmented analysis.

Academic research on visual–inertial SLAM and sensor fusion was rapidly maturing, making its way into wearable formats. By the late 2010s, algorithms like VINS-Mono could tightly integrate camera and IMU data for reliable tracking in real time, and ORB-SLAM3 demonstrated accurate visual–inertial SLAM with monocular or stereo cameras [36]. These advances directly feed into helmet systems; a helmet can now leverage an IMU (often already present in headsets for orientation) alongside the camera to achieve far more robust localization, even under fast head movements or low-texture scenes. It showed ORB-SLAM3’s visual–inertial mode to be 5–10× more accurate than pure vision in challenging scenarios. We also see multi-camera helmets being proposed to capture full 360° views. A notable patent in 2019 by Fiala described a helmet with a multi-camera array covering the entire spherical view, combined with fiducial marker tracking for AR applications [37]. By the early 2020s, the concept of a “smart helmet” had expanded [38]: it now encapsulates devices with multi-modal sensors (visual, thermal, gas, etc.), on-board processing (sometimes cloud-connected), and advanced software (VSLAM, object recognition, and communication tools).

## 2. Methodology

### 2.1. Search Strategy and Paper Selection Criteria

To capture the relevant literature for this review, we employed a multi-pronged search strategy as seen in [39,40]. We focused on two major scientific databases (Scopus and IEEE Xplore), queried using keywords such as

I. “smart helmet” OR “wearable AR helmet”.
II. “helmet camera SLAM” OR “visual SLAM inspection”.
III. “wearable vision system” OR” industrial augmented reality”.

And combinations thereof. Given the interdisciplinary nature of the topic, our search spanned domains of robotics, computer vision, construction engineering, and occupational safety. We also searched patent databases (e.g., Google Patents) for patents with terms like “smart helmet”, “helmet camera system”, and “helmet AR”, which yielded insight into commercially driven innovations.

**Figure 1:**
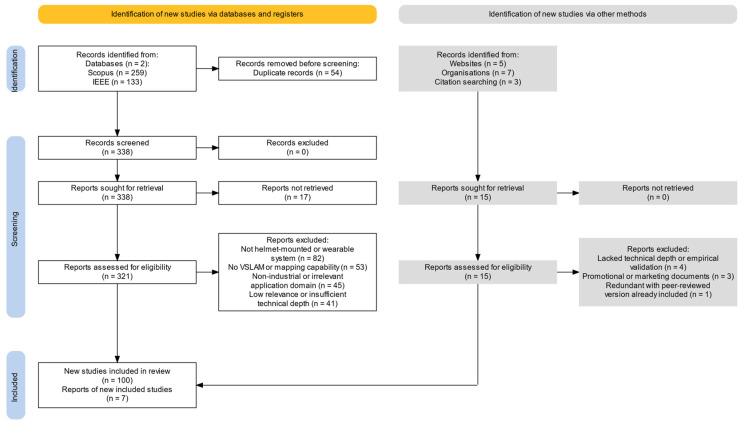
PRISMA flow diagram of the literature search and selection process.

To ensure high relevance and quality, we followed the PRISMA guidelines to document and structure our paper selection. Figure 1 presents the flow diagram, which visually summarizes the identification, screening, eligibility, and inclusion stages of our literature review covering both white and gray literature sources. Our initial search (spanning publications from 2015 to 2025) yielded over 392 papers, and after removing duplicates, we were left with 338.

During the screening process, a number of reports were excluded for various reasons. For scientific articles and conference papers, the following exclusion criteria were applied:

- Not helmet-mounted or wearable: excluded if the focus was not on helmet-mounted or wearable systems.
- No VSLAM or mapping capability: excluded if the work did not address visual SLAM or relevant mapping functionalities central to this review.
- Non-industrial or irrelevant application domain: excluded if focusing solely on non-industrial domains (e.g., gaming and recreational VR/AR) unless they introduced transferable technologies.
- Insufficient technical or methodological depth: excluded if lacking empirical results, technical reproducibility, or in-depth methodological description.

Gray literature sources were screened using the following additional criteria:

- Promotional or marketing focus: excluded if the material was primarily promotional, lacked objectivity, or was produced for marketing purposes without substantive technical content.
- Lack of empirical or practical evidence: excluded if the source did not provide real-world deployment evidence, technical results, or data relevant to inspection/maintenance use cases.
- Redundant with peer-reviewed publication: excluded if peer-reviewed information was available.

We included a limited number of industry reports and product whitepapers to illustrate real-world adoption, such as Real Wear devices, or use cases such as RINA’s marine inspections using the Kiber Helmet [41,42].

After filtering, about 100 sources were identified as highly relevant. Each was reviewed in detail, and from these, we extracted technical details, experimental results, and key insights.

The sections that follow will show a brief discussion of the classification framework and performance evaluation metrics for VSLAM-enabled helmet systems.

### 2.2. Classification Framework for Technologies

Table 1 shows the organization of the diverse literature classification framework. It shows the adopted classification of the three high-level facets, which are camera system topology, VSLAM and sensor fusion techniques, and finally application and development, while Table 2 shows the taxonomy framework for the three high-level facets with examples.

Evaluating the performance of VSLAM-enabled helmet systems requires considering both technical SLAM metrics and application-specific metrics, as can be seen in Table 3.

## 3. Camera System Typologies

Helmet-mounted vision systems can employ different camera configurations, each with its own technical implications for SLAM and inspection capabilities. We identify three main typologies: monocular, stereo, and omnidirectional (360°) camera systems. This section reviews each type, discussing their specifications, implementations in research and industry, strengths and limitations for infrastructure inspection, and how they perform within VSLAM frameworks.

### 3.1. Monocular Camera Systems

A monocular system uses a single camera (or single camera module) on the helmet. This is the simplest hardware configuration, often leveraging a forward-facing RGB camera mounted near the visor or on the side of the helmet. Technically, monocular SLAM is well-studied; it uses a single video stream to both track motion and infer environment structure up to scale [71]. Many early smart helmet prototypes started with monocular cameras due to their low cost, small size, and ease of integration [72,73]. For instance, the Omni Vision patent (2004) integrated a single CMOS camera in a helmet primarily to capture and transmit video [28]. Modern industrial examples include certain AR headsets attached to helmets (like early versions of HoloLens or RealWear devices) [74], which have a single main camera for video streaming and documentation. Table 4 gives a summary of Monocular Camera Typologies with details.

### 3.2. Stereo Vision Systems

Stereo camera systems consist of two cameras (typically a matched pair) mounted on the helmet with a fixed baseline between them, enabling binocular vision and direct depth perception through triangulation [76]. Many modern AR headsets and some smart helmets use stereo vision as it provides the system with a sense of 3D structure akin to human eyes. For example, the DAQRI smart helmet was reported to include “four HD cameras for 360° awareness” and a “3D depth sensor”, effectively using multiple stereo pairs to cover all directions, with Intel RealSense technology providing active stereo depth [77]. Another example is Microsoft’s HoloLens (integrated into some industrial hardhats), which employs stereo gray cameras for environment tracking along with a depth sensor [78]. In research, stereo helmets have been built by mounting calibrated camera pairs on the front or sides of a helmet to achieve real-time depth mapping of the environment [79]. A detailed summary of the stereo vision systems′ topologies can be seen in Table 5 below, highlighting depth perception and accuracy, notable implementations, strengths, limitations, and VSLAM characteristics of the stereo vision system

### 3.3. Omnidirectional (360°) Camera Systems

Omnidirectional vision systems provide a full 360° view of the environment around the helmet, either in the horizontal plane or full spherical coverage (360° horizontal and 180° vertical). This can be achieved by multiple cameras arranged to cover all directions (multi-camera array) or by using specialized optics like fisheye lenses or catadioptric mirrors to capture a wide field in one image [88]. On a helmet, achieving true spherical vision often means mounting several cameras (e.g., front, back, sides, and maybe top) and stitching their outputs into a panoramic view. Some research prototypes have used catadioptric cameras (a camera pointed at a curved mirror) mounted atop a helmet to obtain omnidirectional images [89]. The impetus for omnidirectional systems is to ensure that no feature or object around the user goes unobserved. This is very useful for SLAM (minimizing the loss of tracking) and for situational awareness (the user or remote observers can see everything around, not just where the helmet is facing) [90]. The full summary, explaining the details of typical configurations, image stitching and processing, complete environment capture benefits, notable VSLAM implementations, challenges specific to 360° systems, VSLAM performance, and the inspection and field use benefits of omnidirectional (360°) camera typologies can be seen in Table 6.

### 3.4. Comparative Analysis

Each camera typology offers distinct advantages and faces unique limitations. Here we compare them across several dimensions relevant to industrial deployment

Table 7 summarizes these comparisons. Generally, it can be observed that monocular helmets win on simplicity, cost, and ease, but require more algorithmic aid (like inertial fusion) and careful movement to obtain complete data. Stereo helmets hit a middle ground, giving depth and better accuracy upfront, which improves reliability and measurement utility with moderate added complexity. Omnidirectional helmets provide maximum data and robustness, at the cost of complexity in hardware and software; they shine in specialized scenarios requiring that level of detail. It is worth noting that some hybrid approaches exist too: for instance, a helmet might have a front stereo pair and an additional rear monocular camera, giving depth to what is ahead and at least visual coverage behind. Such hybrid configurations can be seen as a compromise to achieve near-360 coverage without full panorama stitching.

**Figure 2:**
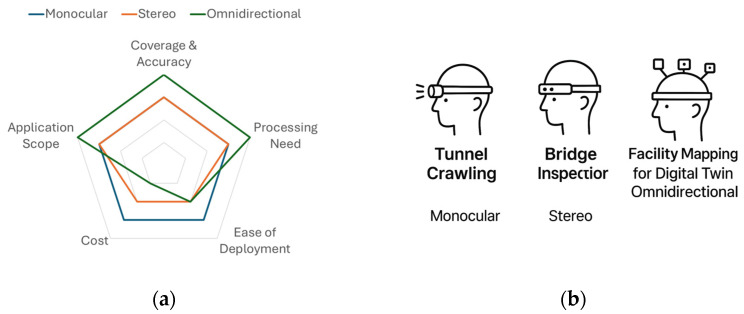
(**a**) Key trade-offs among three camera systems: monocular, stereo, and omnidirectional, (**b**) commonly used in industrial inspection and mapping tasks.

Figure 2 presents a comparative estimation of three common camera system configurations, monocular, stereo, and omnidirectional—frequently employed in helmet-mounted VSLAM systems for industrial inspection and mapping tasks. The radar chart in subfigure (a) synthesizes the performance across five critical criteria derived from the peer-reviewed literature: coverage and accuracy, processing need, ease of deployment, cost, and application scope. These criteria were selected to reflect both technical capabilities and real-world usability in constrained and dynamic industrial settings.

Omnidirectional systems demonstrate a superior performance in coverage and accuracy as well as application scope, owing to their ability to capture full 360° imagery and support rich environmental reconstruction. However, this advantage comes at a cost: their deployment on wearable platforms often requires multiple cameras and complex synchronization, which increases both computational load and system cost. Consequently, they rank lowest in terms of ease of deployment and affordability.

At the opposite end of the spectrum, monocular systems exhibit excellent ease of deployment and cost-efficiency, making them attractive for lightweight, mobile scenarios. Their lower scores in coverage and accuracy stem from a limited field of view and the need for continuous motion to construct complete environmental maps. Stereo configurations occupy an intermediate position, offering a compromise between spatial accuracy and deployment feasibility. Their dual-camera setup supports effective depth estimation while avoiding the bulk and complexity of omnidirectional rigs.

The scenario-based mapping shown in subfigure (b) illustrates how these trade-offs translate into real-world use. In confined environments such as tunnels or pipelines, monocular systems are preferred due to their compact form and minimal processing requirements. Bridge inspection tasks, which demand accurate 3D measurement and structural assessment, benefit from stereo vision’s depth-sensing capabilities. For large-scale facility mapping, particularly for digital twin applications, omnidirectional systems offer comprehensive environmental coverage and contextual awareness, despite higher deployment complexity.

Together, these visualizations underscore that no single camera configuration universally outperforms the others. Instead, optimal system selection hinges on the operational context, balancing practical constraints with performance goals such as localization precision, data fidelity, and system robustness. As the next sections will demonstrate, advancements in VSLAM algorithm design and sensor fusion strategies are increasingly mitigating the limitations of simpler camera setups, enabling a robust performance even in challenging inspection scenarios.

## 4. VSLAM Technologies and Implementations

To convert raw video data from helmet-mounted cameras into actionable information, such as maps, accurate localization, and environmental insights, advanced algorithms are required. Visual Simultaneous Localization and Mapping (VSLAM) lies at the heart of smart helmet systems, enabling real-time trajectory estimation and environmental mapping. This section provides an overview of major VSLAM algorithm categories, a comparative analysis of their strengths and weaknesses, and reviews implementation strategies specifically adapted to wearable inspection contexts.

### 4.1. Core VSLAM Algorithm Categories

The fundamental approaches used in wearable VSLAM are summarized in Table 8, highlighting the characteristics, example algorithms, benefits, and key considerations.

These algorithmic approaches continue to evolve as wearable platforms and sensor suites become more sophisticated. Feature-based and direct methods remain the mainstays for many commercial and research systems, but hybrid and deep-learning-augmented methods are gaining traction as hardware capabilities advance.

### 4.2. Algorithm Impact and Trends (2015–2025)

Building upon the systematic literature review described earlier in Section 2.1, data from the final corpus was analyzed to determine how the impact of each algorithm category evolved over the past decade. The findings, shown in Table 9 and visualized in Figure 2, represent normalized annual impact scores (0–10) based on frequency of adoption, citation trends, and benchmark performances (KITTI, TUM RGB-D, and EuRoC MAV).

**Figure 3:**
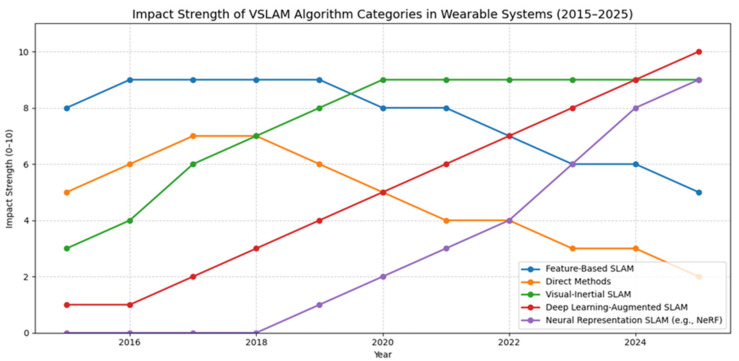
Impact strength (on a scale from 0 to 10) of various VSLAM algorithm categories in wearable systems over the last 10 years (2015–2025).

Figure 3 shows a plot of the past decade on how the landscape of VSLAM (Visual Simultaneous Localization and Mapping) algorithms for wearable systems has undergone a significant transformation, marked by evolving strengths across various algorithm categories. Initially, feature-based SLAM methods such as ORB-SLAM held dominant influence, particularly between 2015 and 2018. Their robustness, relatively low computational demands, and compatibility with mobile hardware made them the go-to choice for early smart helmets and AR devices. As wearable environments grew more dynamic and demanding, the limitations of feature-only methods, particularly under challenging lighting or texture conditions, led to a gradual decline in their impact. By 2025, while still used in lightweight systems, their influence will have tapered.

Direct methods like LSD-SLAM and DSO saw a moderate rise around 2017 due to their ability to operate in low-feature environments by leveraging image intensity gradients. However, their susceptibility to lighting variations, lack of robustness during fast motion, and computational intensity restricted their wider adoption in wearable platforms. As a result, their impact steadily declined in subsequent years. The integration of visual–inertial SLAM has proven to be one of the most impactful advancements, particularly from 2018 onward. Systems that combine camera data with IMU readings like VINS-Mono, OKVIS, and ORB-SLAM3 in visual–inertial mode offer significantly improved stability, scale observability, and drift reduction. In wearable applications, where rapid head movements and temporary vision occlusions are common, visual–inertial fusion has become essential. Since 2020, it has remained one of the most influential SLAM approaches in real-world helmet and AR system deployments. Parallel to sensor fusion advancements, deep-learning-augmented SLAM began gaining traction after 2018. By incorporating learned features, monocular depth prediction, and semantic segmentation, these approaches improved SLAM robustness in texture-poor or dynamic environments. Though initially hindered by computational constraints, the rise in mobile AI accelerators has enabled more on-device inference, allowing wearable systems to benefit from the adaptability and resilience of AI-enhanced pipelines. By 2025, deep-learning-based SLAM is projected to be the most impactful category, especially in complex inspection and industrial scenarios.

According to [104], neural representation approaches such as Neural Radiance Fields (NeRF) have recently emerged as powerful tools for generating dense, photorealistic 3D reconstructions. While not yet widely adopted for real-time SLAM on wearable devices, their role in post-processing and digital twin generation is rapidly expanding. These methods are especially promising for environments requiring high-fidelity documentation, such as tunnels, heritage sites, or industrial facilities. From 2022 to 2025, their impact has grown significantly and is expected to play a central role in future mapping pipelines. Therefore, it can be said that VSLAM in wearable systems has transitioned from classical, handcrafted methods to more integrated, sensor-rich, and learning-based paradigms. Visual–inertial SLAM remains a core technology for real-time tracking, while deep learning and neural scene representations are shaping the future of mapping and spatial understanding in wearable contexts.

### 4.3. Sensor Fusion Approaches for Enhanced Robustness

To overcome the limitations of purely visual approaches, wearable VSLAM often integrates complementary sensors to enhance robustness, accuracy, and reliability. Table 10 summarizes the sensor fusion strategies deployed in helmet systems, including their practical implications:

- Visual–inertial fusion (Camera + IMU): Widely regarded as the gold standard, combining visual data with inertial measurements significantly reduces positional drift, enhances robustness to rapid head movements, and maintains tracking during brief visual occlusions. However, this fusion demands precise calibration and careful sensor synchronization, crucial for high-performance outcomes.
- Visual–LiDAR fusion: LiDAR sensors provide an accurate geometric structure even under poor visibility, greatly enhancing spatial accuracy. While extremely beneficial for high-fidelity maps (digital twins), LiDAR’s cost, weight, and energy consumption make it practical mainly in specialized industrial helmets or backpack-mounted configurations.
- Visual–GNSS fusion: integrating GNSS (GPS/RTK) positioning is beneficial for georeferencing outdoor inspections, limiting drift over extensive areas. However, GNSS accuracy degrades indoors or in covered structures, limiting widespread use without supplemental positioning methods.
- Additional sensors (thermal, barometer, and magnetometer): Sensors like thermal imaging help inspections in low-visibility conditions (smoke and darkness), while barometers and magnetometers support vertical and directional orientation. Despite niche benefits, environmental interferences such as magnetic distortions or thermal-image texture limitations require careful consideration in practical deployment.

### 4.4. Processing Architectures and Computational Strategies

The computational demands of real-time VSLAM in wearable contexts necessitate the thoughtful selection of processing architectures. Table 11 reviews these architectures and their trade-offs:

- Edge computing: On-device processing provides immediate, latency-free operation critical for safety-sensitive tasks. Examples like HoloLens use specialized hardware (CPUs, GPUs, ASICs) optimized for energy efficiency. However, battery life, heat management, and device bulk remain significant engineering challenges.
- Cloud or remote processing: Offloading computation to cloud infrastructures enables the use of sophisticated algorithms (deep learning, photogrammetry). Systems such as the Kiber Helmet demonstrate practical use in remote-expert scenarios. However, cloud reliance introduces latency issues, data security concerns, and dependency on reliable network connectivity.
- Hybrid architectures (edge–cloud synergy): Combining local edge processing with remote cloud computation strikes a balance, performing real-time tasks on-device and leveraging cloud capabilities for complex analysis or data-intensive tasks. This requires sophisticated partitioning strategies and robust synchronization mechanisms to handle real-world operational constraints effectively.
- Hardware acceleration and optimization: GPUs, DSPs, and custom processors significantly accelerate real-time VSLAM tasks, enabling low latency and reduced power consumption. However, integrating specialized hardware involves complexity in software development, cost considerations, and challenges in device integration.

Recent advances in edge AI [105,106] are beginning to transform how wearable systems handle VSLAM. Instead of relying solely on traditional embedded processors, newer helmet platforms are now incorporating dedicated AI accelerators, such as NPUs (Neural Processing Units) or optimized deep learning inference engines, directly into the device. This allows deep neural networks used for tasks like feature extraction or object recognition to run locally, with minimal latency and reduced reliance on cloud resources. These edge AI chips are designed for low power consumption, making them ideal for wearable platforms that demand real-time processing without sacrificing battery life. Integrating edge AI into smart helmets opens the door to more intelligent, context-aware decision making in the field, even when internet access is limited or unreliable.

## 5. Industrial Implementations

Having reviewed the technological underpinnings, we now turn to real-world deployments of smart helmets with integrated vision systems. This section examines key infrastructure inspection workflows, bridges and structural assets, industrial plants, underground infrastructure (e.g., tunnels, mines, and sewers), and power generation facilities that benefit from smart helmet adoption.

Table 12 outlines the typical inspection methods for each domain, highlighting the enhancements enabled by smart helmets and their associated benefits.

To assess the transformative impact of these systems, we analyzed the academic literature and industry reports from the last decade (2015–2025), focusing on how smart helmets enhance three core capabilities:

1. Spatial awareness—helping workers understand their position in complex or GPS-denied environments.
2. Augmented visualization—overlaying relevant data in the user’s field of view.
3. Automation—providing guided procedures, live alerts, or enabling remote expert support.

Using a rating scale from 1 to 5, where 5 indicates high impact, we scored each domain based on the relevance and effectiveness of smart helmet features. The results are shown in Table 13 and visualized in Figure 3.

**Figure 4:**
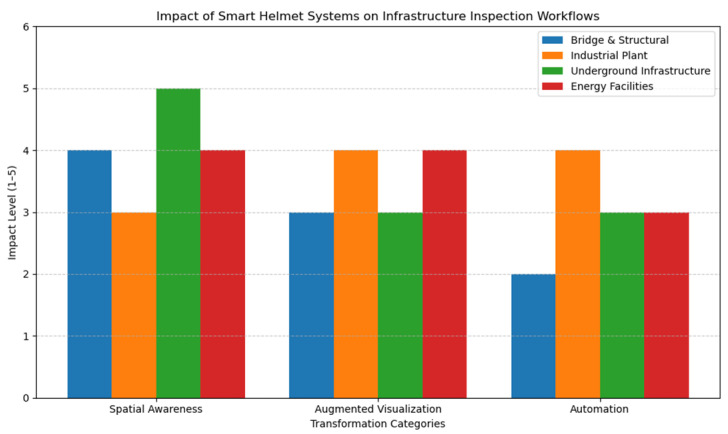
The transformative impact of smart helmet systems across four key infrastructure inspection domains.

Figure 4 illustrates how smart helmet technologies are reshaping inspection practices. Spatial awareness shows the highest impact across all domains, particularly in underground infrastructure (scoring 5), where GPS-denied and hazardous conditions necessitate precise real-time localization. Augmented visualization is notably effective in industrial and energy environments (both scoring 4), where overlaid data enhances situational understanding. Automation sees its strongest effect in industrial settings (score of 4), reflecting the use of smart helmets for procedural guidance, sensor alerts, and remote support. While bridge inspections benefit most from spatial awareness, their automation scores remain lower due to the manual nature of structural assessments.

Following this impact assessment, Table 14 presents a series of illustrative industrial implementations demonstrating real-world implementations across various industries. These examples show measurable improvements in safety, inspection accuracy, and operational efficiency.

## 6. Challenges and Limitations

Despite the promise and demonstrated benefits of VSLAM-enabled smart helmets, there are several Challenges and Limitations hindering their widespread adoption and optimal performance. These span technical issues (hardware and algorithm constraints), human factors (usability and safety for the wearer), and broader regulatory or standardization concerns. Recognizing these challenges is important for guiding future improvements and managing expectations in deployment. Table 15 below highlights the key challenges faced by VSLAM. These challenges were divided into three main categories based on the findings made from the review.

One often overlooked challenge in deploying VSLAM-enabled smart helmets is the ethical dimension, especially around privacy and surveillance. These systems typically involve continuous video capture, environmental scanning, and sometimes biometric data collection (e.g., eye tracking or physiological monitoring). In industrial settings, this can raise concerns among workers about being monitored or recorded without consent and may also conflict with data protection regulations. As noted in [109,110], organizations must take steps to ensure transparent use policies, secure data handling, and, where possible, the anonymization or on-device processing of sensitive information to reduce privacy risks. Designing helmet systems with privacy by design, not as an afterthought, will be crucial for broader acceptance and ethical compliance.

Table 15 highlights a comprehensive set of challenges hindering the adoption of VSLAM-enabled smart helmets across three broad categories: technical, human, and regulatory. Technically, the most pressing issues include short battery life (2–3 h), insufficient processing power for real-time complex tasks like AI or 3D reconstruction, and environmental vulnerabilities such as dust, lighting, or vibration, which impair sensor accuracy. Additionally, the large volume of data generated poses storage, transmission, and privacy challenges, while SLAM algorithms struggle in degraded or feature-poor environments, often requiring fallback systems. Connectivity in remote industrial zones further complicates real-time operations dependent on cloud services. From a human perspective, helmet discomfort, cognitive overload from excessive AR content, difficult user interfaces in noisy environments, and steep training requirements can limit user acceptance. Cultural resistance due to fears of surveillance or job replacement adds another layer of complexity. Regulatory issues also pose significant barriers, including the high cost and time needed for safety certifications, legal concerns over continuous video/audio recording, and a lack of standardization for AR data formats. These multifaceted challenges highlight the need for holistic solutions that address not only the technology itself but also human factors and institutional frameworks to ensure successful deployment and integration in industrial settings.

## 7. Collaborative and Multi-Agent Systems

**Figure 5:**
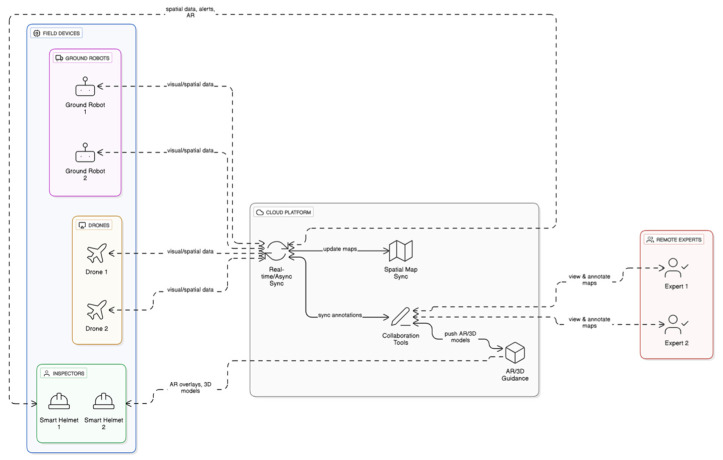
Network diagram for Collaborative and Multi-agent Systems.

Figure 5 shows the network diagram for Collaborative and Multi-agent Systems in smart helmet technology. The network illustrates a dynamic ecosystem where multiple inspectors, drones, ground robots, and remote experts are interconnected through a shared cloud platform. In this system, each smart helmet collects spatial data and communicates with other agents in real time or asynchronously when connectivity permits. Drones and robots contribute by scanning hard-to-reach or hazardous areas, feeding visual and spatial information into the shared map. Remote experts can view these maps and provide live AR guidance, such as virtual annotations or 3D models that helmet users see in their display.

A promising area for future exploration is the development of multi-user or collaborative SLAM systems [46,111]. In large or hazardous environments, such as underground mines, power stations, or disaster zones, multiple workers may be navigating the same space with helmet systems. Enabling these devices to share maps, localize each other, or fuse mapping data in real time could dramatically improve efficiency and safety. For example, if one helmet detects a blocked passage or hazard, that information could be instantly shared with others nearby. While collaborative SLAM has seen early-stage research in robotics, applying it to wearable VSLAM in the field introduces new challenges around synchronization, network stability, and privacy, but also represents an exciting leap forward in operational coordination.

The centralized cloud platform acts as the integration hub, synchronizing maps, annotations, and decision-making tools across all users. The result is a highly coordinated and intelligent inspection workflow where data is pooled, expertise is shared instantly, and inspection efficiency is significantly increased. This visualization reinforces how collaboration transforms smart helmets from standalone tools into part of a scalable, connected system capable of real-time spatial intelligence and cooperative problem solving.

## 8. Conclusions and Recommendations

The integration of VSLAM-enabled technologies in smart safety helmets for industrial infrastructure inspection represents a promising and rapidly maturing field that addresses critical challenges in workplace safety, inspection efficiency, and data quality. The convergence of advanced computer vision algorithms, robust sensor fusion techniques, and wearable computing platforms has created unprecedented opportunities for enhancing human capabilities in hazardous and complex industrial environments. However, successful deployment requires careful consideration of technical limitations, human factors, and organizational readiness, factors that collectively determine whether these promising technologies can achieve widespread industrial adoption and deliver their anticipated benefits in practice.

### 8.1. Research Question Synthesis

This review systematically addressed four key research questions about VSLAM-enabled smart safety helmets for industrial infrastructure inspection:

RQ1 (Technology Characterization): how can current helmet-mounted vision and mapping technologies be examined and categorized?

Our analysis identified three primary Camera System Typologies—monocular, stereo, and omnidirectional, each with distinct trade-offs in cost, complexity, and capability (Section 3). We categorized VSLAM approaches into four main types: feature-based methods (dominant in early systems like ORB-SLAM), direct methods (better for low-texture environments), hybrid approaches (combining multiple techniques for enhanced robustness), and emerging deep-learning-enhanced methods (showing promise but limited current field deployment). Sensor fusion strategies were classified into visual–inertial (most mature and widely adopted), visual–LiDAR (high accuracy but costly and power-intensive), visual–GNSS (for outdoor georeferenced applications), and multi-modal approaches incorporating thermal and environmental sensors. The analysis revealed a clear technological evolution from simple recording devices to sophisticated sensor-fused platforms capable of real-time environmental understanding and spatial intelligence.

RQ2 (Performance Assessment): how well do these systems perform across different industrial inspection scenarios?

Our estimation across four key domains—bridge/structural inspection, industrial plants, underground infrastructure, and power generation facilities—highlights important benefits (Section 5). Underground infrastructure showed the highest impact for spatial awareness (5/5 rating), where GPS-denied environments benefit most from SLAM-based localization. Industrial plants and power facilities benefited most from augmented visualization and automation capabilities (4/5 ratings each), enabling real-time overlay of operational data and remote expert guidance. However, performance varies significantly with environmental conditions, with VSLAM systems struggling in low-texture, dynamic lighting, or feature-poor scenarios without appropriate sensor fusion support.

RQ3 (Implementation Analysis): what challenges affect widespread adoption, and what are the best practices?

Three major challenge categories were identified (Section 6): Technical challenges (battery life limitations of 2–3 h, processing power constraints, and environmental robustness requirements), human factors (ergonomic discomfort, cognitive load management, extensive training requirements, and cultural resistance to surveillance technology), and regulatory issues (safety certification complexity, data privacy compliance, and lack of industry standardization). Proposed best practices for successful deployment include involving end-users in UI/UX design processes, starting implementations in stable indoor environments, ensuring robust connectivity or comprehensive offline capability, providing extensive training programs tailored to diverse skill levels, and establishing clear data governance policies with transparent usage guidelines. Research demonstrates that involving end-users throughout the design and development phases creates more usable equipment and systems [112], with worker involvement in safety-related decision making playing an important role in reducing occupational injuries and achieving safety ownership [113]. The most critical success factor identified was addressing human acceptance through demonstrable value delivery and trust-building rather than focusing solely on technical capabilities.

RQ4 (Future Directions): what are the current research gaps and priority areas for development?

Key research gaps include the absence of standardized benchmarks for helmet-based VSLAM evaluation, limited long-term user acceptance studies in industrial settings, an insufficient analysis of unsuccessful deployments and failure modes, and a need for more robust SLAM algorithms under challenging environmental conditions. Priority development areas encompass energy-efficient edge AI processing architectures, improved sensor fusion techniques for harsh industrial environments, semantic SLAM capabilities for automated defect recognition, collaborative multi-helmet mapping systems, and seamless integration with digital twin workflows. The field is transitioning from proof-of-concept demonstrations toward mature industrial tools, requiring focus on system reliability, industry standardization, and total cost of ownership optimization rather than pure technical advancement. Techniques such as geometry-aware 3D learning for semantic segmentation and precise feature detection [114] or compressed sensing methods leveraging dual-frequency LiDAR [115] offer promising pathways to increase the accuracy and efficiency of spatial mapping in future helmet-mounted VSLAM systems.

This research reveals that while VSLAM-enabled smart helmets have the potential to transform industrial inspection, successful deployment requires holistic consideration of technical capabilities, human factors, and organizational readiness. The technology is quickly maturing beyond research prototypes to viable industrial tools; however, widespread adoption depends on addressing integration challenges and demonstrating clear return on investment in real-world operational environments.

### 8.2. Strategic Implementation Guidance and Recommendations

Addressing these challenges requires a comprehensive approach that balances immediate implementation needs with longer-term technological development. Based on our systematic analysis, we identified ten critical categories, summarized in Table 16, where targeted actions, in our opinion, can accelerate successful deployment and advance the field toward mature, reliable industrial applications.

The implementation strategy should recognize that organizations need immediate, actionable guidance for current technology adoption while researchers require clear priorities for advancing the field’s capabilities.

## Figures and Tables

**Table 1:** Classification framework with taxonomy of three high-level facets.

Facet	Description	Relevant Reference
Camera System Typology	Categorizes helmet-mounted vision systems into monocular, stereo, and omnidirectional types based on camera configuration. Influences VSLAM algorithm choice and depth perception.	[43,44,45]
VSLAM and Sensor Fusion Techniques	Classifies SLAM algorithms and sensor fusion techniques (e.g., visual–inertial and LiDAR fusion). Covers processing architectures like edge computing and cloud offloading.	[46,47,48,49]
Application and Deployment	Categorizes industrial applications: inspections, maintenance, AR guidance, and remote collaboration. Maps combinations of camera systems and SLAM to domains.	[50,51,52]

**Table 2:** Taxonomy framework for subcategories and examples.

Facet	Subcategory	Description	Example from References
Camera System Typology	Monocular	Single forward-facing camera	AR headset using monocular camera [53]
	Stereo	Dual cameras for depth estimation	Stereo vision in medical AR applications [54]
	Omnidirectional	360° coverage with multiple or fisheye lenses	Panoramic vision sensors for navigation [55]
VSLAM and Sensor Fusion	SLAM algorithms	Feature-based, direct, and deep learning SLAM	Learning-based VSLAM [56]
	Sensor fusion	Visual–inertial, LiDAR, and GNSS/IMU	LiDAR + vision integration [57]
	Processing architectures	On-device vs. cloud offloading	Edge computing for real-time SLAM [58]
Application and Deployment	Industrial sectors	Construction, plants, and tunnels	Construction monitoring with helmet-enabled vision [59]
	Use cases	AR guidance, fault detection, and inspection	Detection and guidance in plant inspection [60]

**Table 3:** Performance evaluation metrics for VSLAM-enabled helmet systems.

Evaluation Category	Metric Type	Description	Example/Typical Values	Reference
SLAM Accuracy and Robustness	ATE/RPE/precision–recall/cloud error	ATE (Absolute Trajectory Error), RPE (Relative Pose Error) measure trajectory drift; cloud-to-cloud and Hausdorff distance measure map fidelity	ATE < 0.1–0.3 m; cloud error few cm like 2–5 cm	[36,61,62,63]
Real-Time Performance	Frame rate, latency, and CPU/GPU usage	Evaluates SLAM throughput and feasibility for mobile hardware; goal is real-time performance on embedded processors	15–30 FPS; <100 ms latency	[64,65]
Environmental Robustness	Qualitative stress conditions	Performance under motion blur, low light, smoke, vibration, etc.	Performance maintained in <20 lux lighting, moderate blur	[66,67]
Human Factors	Usability surveys, ergonomics, and cognitive load	SUS score, operator feedback, and fatigue levels after use	SUS score > 70 (acceptable usability); low fatigue over 2 h	[68,69]
Benchmark Datasets	Dataset usage	Use of EuRoC MAV, WHU Helmet, etc., to evaluate accuracy and robustness	The WHU Helmet dataset yielded <5 cm error	[70]

**Table 4:** Summary of Monocular Camera Typologies.

Category	Details
Technical Specs and Capabilities	- Resolution HD to 4K - Shutter Type: global shutter preferred to reduce motion blur - Field of View (FOV): wide (~120°) beneficial for SLAM; introduces distortion - Form factor compact/shallow cameras (e.g., smartphone-sized) can be embedded in helmet brims - Camera Types: range from webcams to high-end machine vision cameras - Depth Estimation: no direct depth; scale/depth inferred from motion - SLAM Algorithms: feature-based (e.g., ORB-SLAM) and direct methods (e.g., LSD-SLAM) - Aids for Scale: use of known markers or initial pacing distance
Implementations and Use Cases	- Research: The 2012 wearable SLAM by Gutiérrez-Gómez et al. (omnidirectional monocular camera) Smart helmet (camera + barometer + IMU) [75] for mountaineering SLAM - Industry: Live video streaming helmets (e.g., Kiber Helmet) Hardware present for SLAM even if not actively used
Strengths	- Cost and Weight: low, as only one camera and minimal hardware - Comfort: lightweight setup improves wearability - Calibration: simpler than multi-camera setups (intrinsics + possibly extrinsics) - Power Efficiency: lower power draw → longer battery life - Algorithm Maturity: ORB-SLAM and others are well-optimized for single-camera input - Inspection Usability: high-res imagery useful for visual inspection and AI-based analysis - Form Factor: easy to conceal in compact mounts (headlamp style) for unobtrusive appearance
Limitations	- No Absolute Scale: without external reference, only relative scaling possible - Poor Low-Texture Performance: may struggle in blank or repetitive environments - Rotation Issues: pure head rotation provides no parallax (no depth) - Feature Dependence: needs texture/features; poor in low light or featureless spaces - SLAM Drift: can accumulate error over distance or in poor environments - No Depth Perception: cannot directly measure object sizes or distances
VSLAM Performance (Monocular)	- Accuracy: typically 1–2% of distance travelled (e.g., 5 cm drift over 30 m) - SLAM Quality: effective loop closure and mapping in small/indoor environments - Environment Sensitivity: performance degrades in dark/reflective/featureless areas - Algorithms: ORB-SLAM, LSD-SLAM (direct method useful in texture-poor settings) - Commercial Examples: Google ARCore (initially monocular camera + IMU) - Scale Solutions: use of known markers, pacing distance, or inertial fusion for metric

**Table 5:** Summary of a stereo vision system typologies.

Category	Details
Depth Perception and Accuracy	- Primary Advantage: direct metric depth via triangulation from left/right camera images [80] - Depth Estimation: small errors (e.g., ~1% error at 1–2 m range with 10 cm baseline) - Application: ideal for dimensioning tasks (clearances, misalignments, and defect sizing) - Three-Dimensional Measurement: enables point-to-point measurements (e.g., crack length in AR view)
Notable Implementations	- Integrated stereo RGB and thermal cameras for 3D point clouds, georeferencing, and structural alignment [81] - Industrial: “Helmet-based laser scanning + stereo vision” in mines, mapping tunnel geometry in real time with stereo and IMU [82] - Research: researchers adapt existing stereo rigs for helmets to capture depth images and enable on-the-fly measurements [83]
Strengths	High precision in dimensional measurements (acts like a portable 3D scanner) [84] - Robustness: higher robustness and lower drift compared to monocular SLAM (due to depth info) - Textureless Areas: depth recovery via disparity even in low-texture regions - Three-Dimensional Object Detection: enables obstacle avoidance and tripping hazard warnings (based on 3D perception) - Defect Detection: improved crack differentiation (real crack vs shadow) and volume measurements - AR Integration: better alignment of virtual and real-world objects in AR applications (due to absolute scale)
Limitations	- Complexity: requires precise calibration for intrinsic and extrinsic camera parameters [85] - Calibration Drift: can be affected by mechanical shocks or temperature changes - Processing Demand: stereo image processing requires higher computational resources, reducing frame rates or increasing power usage - Baseline Trade-Off: larger baseline yields better depth accuracy at longer ranges, but limits near-field precision and can be physically cumbersome on helmets - Textureless/Featureless Areas: both stereo cameras face challenges in low-light or featureless conditions (similar to monocular issues) - Bulk and Cost: additional hardware increases bulk and cost, with two cameras and potentially an IR projector - Power Consumption: more demanding on power, especially in real-time high-res stereo setups
VSLAM Characteristics	Performance: stereo SLAM systems (e.g., ORB-SLAM2/3) outperform monocular in terms of localization accuracy and map fidelity [86] - Trajectory Estimation: stereo systems can double the accuracy of monocular VSLAM [87] - Dense Point Clouds: stereo systems generate dense point clouds for real-time visualization of 3D structure (e.g., in building scans) - Fusion with IMU: combining stereo with visual–inertial SLAM leads to very stable tracking - Real-Time Mesh Building: stereo depth can be used to build meshes (e.g., KinectFusion with stereo depth as RGB-D source)

**Table 6:** Summary of omnidirectional (360°) camera typologies.

Category	Details
Typical Configurations	- Two Fisheye Cameras: back-to-back for spherical view (similar to consumer 360° cameras) [91] - Three to Six Normal Cameras: overlapping FOV to achieve ~360° coverage - Patented Designs: the authors of [92] proposed outward-facing multi-cam helmet for spherical vision - Catadioptric mirror + camera for full-view display inside helmet [93] - Industrial Use: Insta360 or GoPro Max on hardhats for full-environment capture
Image Stitching and Processing	- Challenges: real-time stitching is compute-heavy; misalignment leads to visual artifacts [94] - SLAM Integration: some SLAM systems process individual camera streams (multi-cam SLAM) - ORB-SLAM3: supports multi-camera input with fixed extrinsics - Fisheye SLAM: some convert to spherical models for SLAM using specialized projections - Calibration Complexity: requires intrinsics + extrinsics for all cameras or accurate mirror modeling
Complete Environment Capture Benefits	- Omnidirectional Advantage: captures entire surroundings, reducing SLAM failure on rotation [95] - Loop Closure: enhanced due to wide-angle/global visibility [96] - Persistent Features: features remain in view longer, minimizing tracking loss - Awareness: can alert user to behind-the-head events (e.g., steam leaks) - Remote Viewers: experts can freely navigate 360° feed without needing user to reposition [97]
Notable VSLAM Implementations	- Fisheye visual–inertial SLAM, improved robustness indoors [98] - A 360° mapping in GNSS-denied environments [70] - Topological SLAM using omnidirectional vision for robust relocalization [99]
Challenges Specific to 360° Systems	- Data Volume: multiple streams = high bandwidth/storage use - Sync Issues: requires hardware synchronization for temporal consistency - Exposure Variance: different lighting conditions per camera complicate processing - Form Factor Risks: exposed cameras/mirrors prone to damage; needs rugged design - Bulk and Cost: more cameras = higher cost and larger form factor - Lighting Inconsistencies: can cause seam visibility in stitched images - Real-Time Constraints: may require resolution/frame rate trade-offs to stay in real time
VSLAM Performance	- Robust Tracking: SLAM continues even if some cameras are occluded - Loop Closure: strong performance due to comprehensive feature capture - Accuracy: sub-centimeter reported in 20 m loops under structured conditions - Mapping Efficiency: full environment mapped in one pass; supports fast inspection - Thermal + RGB: experimental helmets using both modalities for rich defect detection (e.g., hotspots)
Inspection and Field Use Benefits	Wide-Area Documentation: captures scenes behind or beside user - Efficient Surveying: one pass = full data capture of environment - Advanced Applications: enables panoramic thermal maps and geometric overlays - Human-Viewable Output: panoramas for reports (tunnel interiors and plant rooms) - Analytical Potential: object tagging, defect localization, and 3D overlays

**Table 7:** Comparative analysis of camera systems.

Criteria	Monocular	Stereo	Omnidirectional
Mapping Accuracy and Coverage	- Directional view only; limited coverage. - User must move to different angles. - Accurate locally but prone to scale drift. - Adequate for systematic inspections.	- High depth accuracy (~120–150°). - Limited coverage unless more pairs added. - Low trajectory error (<1%).	- Full 360° coverage in one pass. - Best for wide/irregular spaces. - Excellent tracking and map completeness. - High depth accuracy.
Processing Requirements	- Lightest load; single feed. - Runs on smartphone-grade processors at ~30 FPS.	- Double data and disparity computation. - In total, ~15–20 FPS on same hardware.	- High load (e.g., 4× with 4 cams). - May need GPU (e.g., Jetson) or belt unit. - May record for post-processing.
Practical Deployment	- Small, lightweight, easy to mount. - Safest in confined spaces. - Simple to protect.	- Needs fixed baseline mount. - May protrude from helmet. - Moderate complexity.	- Complex integration (multi-units or panoramic). - Risk of imbalance or safety concerns. - More failure points.
Cost–Benefit	- Most affordable (~hundreds USD). - High return for simple tasks. - Ideal for budget scenarios.	- Moderate cost (~2× monocular + sync). - Good balance of cost and depth.	- Most expensive (multi-cams + software). - Best for high-stakes or one-pass mapping (e.g., digital twins).

**Table 8:** Summary of the core VSLAM algorithm categories in wearable systems.

Category	Description	Example Algorithms	Strengths	Challenges/Considerations for Wearables
Feature-Based (Indirect)	Extracts and matches visual features like corners/edges across frames	- ORB-SLAM, ORB-SLAM2/3 - PTAM - Marker-based AR (AprilTags, QR codes)	- Efficient on limited hardware—good in textured, structured environments - Mature graph optimization methods (e.g., bundle adjustment) - Marker-based alignment for stability	- Struggles with motion blur, low-texture scenes - Performance tied to feature quality - Can require adaptation for lighting or scale issues [99,100,101]
Direct Methods	Operates on raw pixel intensities to estimate motion by minimizing photometric error	- LSD-SLAM - DSO (Direct Sparse Odometry)	- Better in low-texture areas - Can be more accurate with photometric consistency	- Sensitive to lighting changes - Can be computationally intensive - Semi-dense/dense mapping needs tuning for mobile hardware
Hybrid Approaches	Combines feature and direct methods or integrates inertial/other sensors	- SVO (Semi-Direct VO) - ORB-SLAM3 with IMU - Learned depth with classical SLAM back-end	- Combines strengths (e.g., robust tracking + fast pose estimation) - Visual–inertial systems improve robustness - Leverages structured scenes (lines and planes) in wearables [102]	- Complexity in integration - Sensor synchronization - May still be compute-heavy for real-time use [82,103]
Deep-Learning-Enhanced	Applies deep networks for features, depth, or end-to-end SLAM	- SuperPoint, LF - Net (learned features) - PoseNet (pose regression)—semantic SLAM systems	- More robust features under tough conditions - Potential for semantic understanding - Can improve relocation/loop closure	- High compute demand - Real-time use on helmets is limited unless offloaded - Cloud dependency poses latency/reliability issues

**Table 9:** Data used for the visualization of impact strength of various VSLAM algorithm categories in wearable systems over the last 10 years (2015–2025).

Year	Feature-Based SLAM	Direct Methods	Visual–Inertial SLAM	DL-Augmented SLAM	Neural Representation SLAM
2015	8	5	3	1	0
2016	9	6	5	1	0
2017	9	7	7	2	0
2018	9	7	8	3	1
2019	9	6	9	4	2
2020	8	5	9	5	3
2021	8	4	9	6	4
2022	7	4	9	7	5
2023	6	3	9	8	7
2024	5	2	9	9	9
2025	4	1	9	10	10

**Table 10:** Summary of sensor fusion approaches in VSLAM for wearable (helmet) systems.

Fusion Approach	Fused Sensors	Key Algorithms/ Systems	Benefits	Challenges/Considerations for Wearables
Visual–Inertial Odometry (VIO)	Camera + IMU (accelerometer + gyroscope)	- VINS-Mono - OKVIS - ORB-SLAM3 (VI mode)	- High-frequency motion prediction - Smooth 6-DoF tracking with low drift - Scale recovery for monocular SLAM	- Requires precise time and spatial calibration - Sensitive to IMU bias/drift without visual correction - Needs synchronization routines and noise modeling
Visual–LiDAR Fusion	Camera + LiDAR (2D or 3D)	- WHU-helmet project	- Accurate structure from LiDAR, rich texture from vision	- Heavy and power-hungry sensors
Visual–GNSS Fusion	Camera + GPS/GNSS (RTK or standard)	- GNSS-tagged SLAM maps - Outdoor inspection systems	- Anchors SLAM maps globally - Limits drift over large areas - Useful for georeferencing and switching indoor/outdoor	- GNSS unreliable indoors - Standard GPS has low accuracy (~3–5 m) - RTK requires base station and adds complexity
Other Sensors (Barometer, Magnetometer, etc.)	Barometer, magnetometer, digital compass	- Altitude tracking in SLAM - Compass-based orientation alignment - Mountaineering helmet research with barometer	- Adds vertical awareness (elevation gain/loss) - Compass can aid map orientation (e.g., aligning to north)	- Magnetic interference common in industrial settings - Barometer only gives relative change, not absolute height - Requires fusion logic and filtering
Thermal/RGB/Depth Fusion	Multi-modal cameras (RGB + thermal, RGB-D)	- Inspection helmets (heat mapping, etc.)	- Enables mapping in low-light/smoke conditions - Thermal data useful for inspection (e.g., heat leaks)	- Thermal images lack visual texture - Not useful alone for SLAM - Needs co-registration with RGB for accuracy
Fusion Level	Varies by system	- EKF for VIO (older) - Tightly coupled graph optimization (modern SLAM) - Loose coupling (parallel visual and LiDAR SLAM)	- Tight fusion improves consistency - Flexible trade-off with computation	- Tight fusion more complex and computationally intensive - Loose fusion simpler but less accurate - Real-time needs may favor lightweight or partial fusion

**Table 11:** Summary of processing architectures for smart helmet systems.

Architecture Type	Key Features	Examples	Benefits	Challenges/Considerations for Helmets
Edge Computing (On-Device)	All processing is conducted on or very near the helmet (CPU, GPU, and SoC in helmet or wearable module)	- Microsoft HoloLens (CPU, GPU, and HPU) - Jetson TX2/AGX Xavier-based helmets - Snapdragon AR devices	- Low latency, real-time feedback - Works offline - Greater reliability for safety-critical use	- Limited by battery, power, and heat— needs efficient, optimized algorithms—bulkier if high compute power is required
Cloud/Remote Processing	Processing is offloaded to cloud or remote servers. Helmet streams data (video and sensor) for processing	- Kiber Helmet (telepresence) - Cloud-based photogrammetry - Remote AI anomaly detection	- Virtually unlimited compute - Enables complex algorithms (e.g., 3D recon and deep learning) - Reduces helmet weight and heat	- Depends on connectivity (WiFi/5G) - Latency may hurt real-time use - Data privacy/security concerns - Not suitable for all environments
Hardware Acceleration	Use of GPUs, DSPs, NPUs, FPGAs, or ASICs to speed up SLAM, vision, and AI tasks	- NVIDIA Jetson GPU - Snapdragon Hexagon DSP—HoloLens HPU (custom ASIC - Google Tango Motion Processor	- Enables real-time performance - Lower power than general-purpose CPU - Specialized acceleration for SLAM or AI	- Often proprietary/hard to program - Adds design complexity - Thermal and integration challenges
Real Time vs. Post-Processing	Trade-off between immediate on-site feedback and later high-fidelity analysis	- Edge SLAM + Cloud Map Refinement - Record-then-process pipelines - ROS-based prototyping (RTAB-Map)	- Flexible workload distribution - Enables high-quality reports later - Balances power use and responsiveness	- Limited real-time capability on low-end edge - Risk of missing real-time feedback in post-only systems
Edge–Cloud Synergy (Hybrid)	Combines local and remote processing for balanced performance and responsiveness	- Live tracking on edge, deep analysis in cloud—Remote expert guidance + local AR - Local anomaly detection + cloud verification	- Efficient use of resources - Can reduce latency for key tasks - Adapts to available network	- Still needs careful task partitioning - Risk of data sync issues - Complexity in system integration
Software Frameworks	Middleware/platforms for managing sensor data and processing pipelines	- ROS (Robot Operating System) - Custom C++/CUDA pipelines	- Modular and extensible - Easier prototyping - Community support (for ROS)	- ROS not always optimized for embedded use - Custom code can be hard to maintain - Must be robust for field use
Power and Battery Optimization	Strategy to manage compute and power load to extend operational time	- Power-saving modes (pause processing when still) - Edge vs. cloud trade-offs for power	- Prolongs battery life—keeps helmet cooler - Supports longer inspections	- Processing must scale with motion/context - Extra logic to manage compute schedule
Security and Privacy	Ensures inspection data is handled securely	- On-premise edge servers - Encrypted cloud channels - Local-only processing	- Data protection in sensitive sites - Compliance with regulations - Prevents leakage of proprietary data	- Limits use of public cloud - Adds cost for secure infrastructure - Needs user authentication, access control

**Table 12:** Infrastructure inspection workflows for several classes of infrastructure.

Inspection Type	Traditional Methods	Smart Helmet Enhancements	Benefits
Bridge and Structural Inspections	Manual visual inspection, rope/lift access, paper notes, handheld cameras	SLAM-based geo-tagged mapping, hands-free video/data capture, AR overlays of crack maps/BIM, voice or gaze-based defect tagging	Increased safety (hands-free), accurate location-based defect logs, overlay of design data, reduced post-processing
Industrial Plant Inspection	Clipboard-based rounds, manual gauge checks, heavy reference manuals	Camera-based OCR of gauges, AR display of machine data, voice-guided procedures, live remote expert support	Faster decision making, reduced cognitive load, real-time alerts, reduced need for expert travel
Underground Infrastructure (Tunnels, Mines, Sewers)	Flashlights, GPS-denied mapping, environmental monitors, manual sketching	SLAM with LiDAR/360° cameras, AR navigation cues, real-time 3D thermal/visual mapping, environmental data overlays	Improved situational awareness, immediate model generation, navigation aid in zero visibility, hazard alerts in context
Power Generation and Energy Facilities	Paper-based records, handheld inspections, physical marking, high-risk confined space entry	Location-aware data capture, AR component labeling, visual overlays of future installations, interior mapping for turbines/boilers	Safer confined space entry, error prevention in maintenance, planning aid for upgrades, objective image logs

**Table 13:** Data used for visualizing the transformative impact of smart helmet systems across four key infrastructure inspection domains.

Transformation Category	Bridge and Structural	Industrial Plant	Underground Infrastructure	Power Generation
Spatial Awareness	4	3	5	4
Augmented Visualization	3	4	3	4
Automation	2	4	3	3

**Table 14:** Illustrative industrial smart helmet/headset implementations.

Industrial Implementation	Application Domain	Helmet/Headset Role	Reported Benefits	Technologies Used
RINA Marine Surveys (Italy) [41]	Marine vessel inspection	Remote expert support through live helmet video and AR markup	Reduction in inspection time, reduced travel cost, improved safety, and accuracy	Kiber 3 Helmet, live video streaming, AR annotations
Boeing Wiring Assembly (USA) [107]	Aerospace manufacturing, harness assembly	AR-guided step-by-step wiring layout on work surface	Reduction in assembly time, near-zero error rate	AR wearable headsets, Visual Task Guidance
WHU Underground Mine Mapping (China) [70,82]	Mining/tunnel infrastructure mapping	Real-time and post-processed 3D SLAM mapping of tunnels	Survey-grade 3D point clouds, mapping performed in minutes vs. hours, centimeter-level accuracy	Helmet with LiDAR, IMU, cameras, and SLAM algorithms
Worker Safety Monitoring (South Korea) [108]	Construction site safety compliance	Real-time detection of hazards, PPE compliance, and environmental monitoring	Near elimination of heatstroke cases, reduction in unsafe behavior, and safety incident prevention	AI-enabled smart helmets, environmental sensors, cameras

**Table 15:** Key challenges faced by VSLAM-enabled smart helmets in three main categories: technical challenges, human factors, and regulatory and standardization issues.

Category	Challenge	Description
Technical Challenges	Battery Life Constraints	High-performance cameras, processors, and transmitters consume significant power, leading to limited battery life (2–3 h), which is insufficient for full work shifts. Solutions include using belt-mounted packs, but that adds a tether and may not be practical in remote settings.
	Processing Power Limitations	Wearable devices have limitations in processing real-time complex algorithms like 3D reconstruction or AI detection, causing trade-offs between functionality and performance (e.g., reduced mapping or slower responsiveness).
	Environmental Factors Affecting Performance	Harsh environments (lighting variability, dust, rain, vibration, and EMI) degrade sensor performance, leading to potential failures in SLAM. Additional ruggedization or fallback sensors (LiDAR and radar) are required but add cost and weight.
	Data Management and Storage	High data volumes (video, 3D scans, and HD images) challenge storage and bandwidth, especially in remote locations. Managing data lifecycle, archiving, and compliance adds complexity. Privacy concerns and encryption are needed.
	Localization in Degraded Situations	SLAM can fail in low-feature or dynamic environments, causing tracking errors or map failures. Solutions include manual reinitialization or fallback systems, but they impact user experience.
	Connectivity in Real-time Systems	Smart helmets dependent on cloud services face network challenges in industrial environments with limited connectivity, requiring solutions for offline autonomy and local networks.
Human Factors	Ergonomics and User Acceptance	Helmets can be uncomfortable (heavy, warm, and restrictive), leading to resistance from users. Balance between comfort, safety standards, and system functionality is essential to encourage adoption.
	Training Requirements	Workers need extensive training to use the system effectively, which may be challenging for non-tech-savvy workers. Designing intuitive interfaces and minimizing learning curves is key.
	Cognitive Load during Operation	AR systems can overwhelm users with excessive information, causing distractions or fatigue. The interface must provide contextually relevant information without causing mental overload.
	User Interface Considerations	Hands-free control via voice or gestures can fail in noisy or restrictive environments. Clear feedback and simple, quick task execution are necessary to avoid user frustration and ensure adoption.
	Cultural and Workflow Changes	Resistance to technology due to concerns over autonomy or surveillance can hinder adoption. Change management, trust-building, and clear communication of the system’s role as an assistant rather than a replacement are critical.
Regulatory and Standardization Issues	Safety Certifications for Industrial Environments	Helmets must meet strict safety standards (e.g., impact resistance and ATEX for explosive environments). Certification processes are expensive and time-consuming, which limits deployment in hazardous environments.
	Data Privacy and Security Concerns	Continuous data collection (video/audio) raises privacy concerns. Companies must establish clear data usage policies and secure communication to avoid misuse and comply with privacy laws.
	Integration with Existing Safety Protocols	Smart helmets must integrate with traditional safety protocols (e.g., lockout–tagout) and may need regulatory changes for digital tools to be accepted as equivalent to physical safety measures.
	Lack of Industry Standards for AR Data and Interoperability	Lack of standardization for AR inspection data formats leads to vendor lock-in and interoperability challenges. Industry guidelines are evolving but are not yet fully established.
	Ethical and Legal Issues of Recording	Legal implications of continuous video/audio recording, especially without consent, require clear policies and user consent. Intellectual property protection and privacy concerns for workers must be addressed.
	Workforce Acceptance and Labor Relations	Resistance to helmet technology due to concerns over surveillance or loss of autonomy requires thoughtful communication and potential labor agreements to ensure trust and positive adoption.

**Table 16:** Recommendations and future research direction.

Category	Recommendations	Future Research Directions
SLAM and Perception	Use proven SLAM solutions (e.g., ORB-SLAM3) and begin with stable indoor deployments	Develop robust SLAM that adapts to challenging conditions using event cameras, radar, and sensor fusion
AI and Semantic Analysis	Start integrating pre-trained models for basic object detection and labeling	Build semantic SLAM systems that understand environments (pipes, defects, etc.) and support automated defect tagging
Human–Computer Interaction	Involve inspectors in UI/UX design and train them on AR usage effectively	Study AR content delivery to reduce distraction and cognitive load, personalize content via adaptive AI
Hardware and Integration	Select ergonomic helmet designs with reliable battery life; plan for regular calibration	Research miniaturized components, edge AI chips, and seamless AR displays like micro-LED or visor projection
Data and Digital Twins	Centralize data storage and begin fusing helmet outputs with BIM/IoT dashboards	Enable dynamic digital twins updated in real time with wearable and IoT data
Collaboration Tools	Deploy remote expert systems (e.g., RealWear, Kiber) for guidance and training	Research shared spatial maps, swarm SLAM, and AR/VR platforms for team coordination and multi-user annotation
Networking and Infrastructure	Ensure secure, robust connectivity (WiFi or 5G) in inspection areas	Develop decentralized SLAM, compression methods for SLAM data, and robust map merging protocols
Standardization and Policy	Define clear IT and privacy policies; maintain devices and data proactively	Create benchmarks and datasets for helmet-based inspections; collaborate on open standards and IP-aware design
Industry Adoption	Launch pilot projects with clear metrics; document “quick win” successes to justify scaling	Study cost–benefit and impact of helmet deployment in large-scale industrial maintenance cycles
XR for Training	Use AR helmets to assist live tasks; link training to operations when possible	Explore using helmet-collected 3D models for VR-based training scenarios and study training-transfer effectiveness

## Data Availability

The original contributions presented in this study are included in the article. Further inquiries can be directed to the corresponding author.
